# Methylene Blue–Mediated Antimicrobial ​Photodynamic Therapy Against Clinical Isolates of Extensively Drug Resistant ​Gram-Negative Bacteria Causing Nosocomial Infections in Thailand, An *In Vitro* Study

**DOI:** 10.3389/fcimb.2022.929242

**Published:** 2022-07-01

**Authors:** Chankiat Songsantiphap, Jakapat Vanichanan, Tanittha Chatsuwan, Pravit Asawanonda, Einapak Boontaveeyuwat

**Affiliations:** ^1^ Photodermatology Unit, Division of Dermatology, Department of Medicine, King Chulalongkorn Memorial Hospital and Faculty of Medicine, Chulalongkorn University, Bangkok, Thailand; ^2^ Division of Infectious Diseases, Department of Medicine, King Chulalongkorn Memorial Hospital and Faculty of Medicine, Chulalongkorn University, Bangkok, Thailand; ^3^ Department of Microbiology, Faculty of Medicine, Chulalongkorn University, Bangkok, Thailand; ^4^ Antimicrobial Resistance and Stewardship Research Unit, Faculty of Medicine, Chulalongkorn University, Bangkok, Thailand

**Keywords:** photodynamic therapy, methylene blue (MB), antimicrobials, multidrug resistance (MDR), nosocomial infection

## Abstract

**Background/Purpose:**

Some multidrug-resistant gram-negative bacteria as a global threat have been recently prioritized for research and development of new treatments. We studied the efficacy of methylene blue–mediated antimicrobial photodynamic therapy (MB-aPDT) for the reduction of extensively drug-resistant *Acinetobacter baumannii* (XDR-AB) and *Pseudomonas aeruginosa* (XDR-PS) and multidrug-resistant *Klebsiella pneumoniae* (MDR-KP) isolated in a university hospital setting in Thailand.

**Method:**

Two isolates of each selected bacterium were collected, XDR-AB1 and AB2, XDR- PS1 and PS2, and MDR-KP1 and KP2. Three triplicate experiments using various MB concentrations alone, various red light fluences alone, as well as the selected non-toxic doses of MB and fluences of red light combined as MB-aPDT were applied on each selected isolate. The colonies were counted [colony forming units (CFU)/ml]. Estimation of the lethal treatment dose defined as reduction of > 2 log_10_ in CFU/ml compared with untreated bacteria.

**Result:**

There were generally negligible changes in the viable counts of the bacterial suspensions treated with all the MB concentrations (p > 0.05). In the second experiment with the only red light treatments, at fluences higher than 2 J/cm, reduction trend in viable counts across all the isolates was observed. Only for MDR-KP1, however, the lethal dose was achieved with the highest fluence of red light (80 J/cm). With the concentration of MB, 50 and 150 mg/L in the third experiment (MB-aPDT), the greater bacterial reduction was observed in all clinical isolates leading to their lethal viable cell reduction when escalating the light fluence to 80 J/cm.

**Conclusions:**

MB-aPDT evidently killed the selected XDR and MDR-gram negative bacteria. In highly drug-resistant crisis era, MB-aPDT could be a promising option, particularly for local infections and infection complicating chronic wounds.

## Introduction

An initial broad-spectrum empirical antimicrobial treatment is a common practice at least until causative pathogens and their antibiotic susceptibility patterns are determined. As microorganisms can rapidly develop resistance against the widespread use of various antimicrobial agents, this has become a global concern in the past several years, reaching crisis in many settings (Prescott, 2014). Certain infections are rapidly becoming untreatable (Rice, 2008; Bush and Jacoby, 2010; Wang et al., 2020). The term “ESKAPE” (Rice, 2008; Bush and Jacoby, 2010) originally made up of the group of six highly potentially multidrug-resistant (MDR) and virulent bacteria has been known as the leading causes of life-threatening nosocomial infections across the globe. World Health Organization (WHO) recently described a priority list for research and development for new treatments regarding the four most importantly weighted criteria including treatability, mortality, heathcare burden, and 10-year trend of resistance (Tacconelli et al., 2018). Of the ESKAPE pathogens, carbapenem-resistant *A.baumannii* and *Pseudomonas aeruginosa*, and *Klebsiella* spp. have been stratified into the critical priority tier. These three invasive pathogens can cause a wide variety of infections ranging from wound, burn, soft tissue, liver, urinary tract infections, pneumonia to meningitis (Dijkshoorn et al., 2007; Driscoll et al., 2007; Kerr and Snelling, 2009; Ramirez et al., 2020) particularly in neonates, the elderly, and the immunocompronised in a hospital setting.

To fight against the drug resistance crisis, apart from a thrive to develop highly efficient multi-target antibiotics and the discovery of new microbial natural products (NPs) (Hutchings et al., 2019), photoantimicrobials seem to be a promising instant way out of this issue particularly for local skin and soft tissue infections. Methylene blue–mediated photodynamic therapy (MB-PDT) has been known as a powerful monotherapy or adjunct tool to broad-spectrum antimicrobials (Alberdi and Gomez, 2020; Bowornsathitchai et al., 2020). A variety of methylene-blue formulations allow specific delivery of the photosensitizer (PS) to an infected area while sparing adjacent healthy tissue. In addition, the use of economical long-life light sources offers financial affordability to both healthcare providers and patients. Compatibility with other necessary systemic therapies makes MB-PDT nearly universally patient-friendly even for medically complex patients with polypharmacy. Acceleration of wound healing is the other exceptional advantage of MB-PDT over other antimicrobial therapies (Bowornsathitchai et al., 2020) particularly for treating infection complicating chronic wounds.

Despite various factors stated to cause delayed wound healing, infections (Fu, 2005) appear to be a principal contributing factor of chronic wound development in the Middle East and Asia particularly in patients with diabetes (Basu et al., 2009; Jiang et al., 2011), leading to disabling diabetic foot ulcers (Sugandhi and Prasanth, 2014; Tekin et al., 2014; Li et al., 2018; Jouhar et al., 2020; Guan et al., 2021). The greatly diverse and exceptionally dynamic genetic composition of the successful opportunistic pathogens primarily contributes virulence and antibiotic resistance. This genomic diversity has been found not only the strains in different clinically relevant infections but also among ones isolated from different geographic locations (Subedi et al., 2018; Galac et al., 2020; Lebreton et al., 2021; Kiyaga et al., 2022).

Despite many encouraging outcomes of MB-aPDT for MDR-gram positive and negative bacteria reported from many countries, there has been yet no data for the efficacy of MB-aPDT against carbapenem-resistant *A. baumannii* and *P. aeruginosa*, and MDR- *K. pneumoniae* in Thailand. Hence, our objectives were to study the efficacy of MB-aPDT for the reduction of carbapenem-resistant or extensively drug-resistant *A. baumannii* (XDR-AB), carbapenem-resistant or extensively drug-resistant *P. aeruginosa* (XDR-PS), and MDR *K. pneumoniae* (MDR-KP) collected and isolated in a university hospital setting in Thailand.

## Materials and Methods

### Bacterial Strains and Culture Conditions

The study protocol was approved by the Institutional Review Board of Faculty of Medicine, Chulalongkorn University, and conducted under good practice and ethical principles of Declaration of Helsinki 2008. All identified isolates were obtained by Microbiology laboratory, Department of Microbiology, Chulalongkorn University, during June 2019 to August 2020. Two isolates of *A. baumannii* (AB1 and AB2) were collected from sputum and chronic wound infection, respectively, *P. aeruginosa* (PS1 and PS2) from bile and urine, respectively, and *K. pneumoniae* (KP1 and KP2) from sputum. Detailed patients’ profiles and antimicrobial susceptibility pattern of each isolate done according to the Clinical and Laboratory Standards Institute (CLSI) guideline (Weinstein, 2021) were shown in **Supplementary Tables 1** and **2**, respectively. AB and PS were XDR, whereas KP was MDR strains. Each strain was cultivated overnight on trypsic soy agar (TSA) plates at 37°C. A suspension of each strain was then prepared in sterile phosphate-buffered saline (PBS) (pH = 7.4) to McFarland turbidity standard of 2, a concentration approximately of 6 × 10 ^(9)^ to 9 × 10 ^(9)^ colony forming units (CFU)/ml.

### Photosensitizer Preparation

MB (Merck KGaA, Darmstadt, Germany) solution was prepared in normal saline solution (NaCl), filtered-sterilized, and kept in the dark. Any procedures involving MB were performed in a dark room under weak yellow light exposure (λ = 596 nm) to minimize PS activation.

### Effect of MB Concentration on Bacterial Population

The prepared MB was diluted with 0.9% NaCl to give final concentrations of 10, 25, 50, 100, and 150 mg/L. For each concentration, a MB aliquot (500 μl) was added to an equal amount of each bacterial strain suspension and subsequently incubated in the dark for 10 min (MB/bacteria). A dilution of bacterial aliquot with 0.9% NaCl in 1:1 served as negative control. The MB/bacteria mixture suspensions were washed in PBS to remove unbound PS and centrifuged at 13.00×g for 5 min twice until the pellets of bacterial suspension were formed. Cell pellets of each bacterial strain were resuspended in 1 ml of PBS. Then, aliquots (100 μl) in PBS were subsequently transferred into 96-well microtiter plates. Viable bacterial count in CFU/ml was determined after incubation at 37°C overnight.

### Effect of Red Light on Bacterial Population

An aliquot (500 μl) of each aforementioned bacterial strain suspension prepared in sterile PBS without the addition of MB or subsequent incubation in the dark was transferred into a transparent 96-well plate. The microtiter plates were subsequently placed at a constant distance of 4 cm under the illumination head of incoherent red light (peak emission spectrum at 633 nm, 65 mW/cm ^(2)^) from light emitting diodes (HEALITE^®^, Lutronic Corp., Goyang, S. Korea). Irradiations were carried out with the light fluences of 1, 2, 5, 10, 20, 40, and 80 J/cm ^(2)^, which took 0.20, 0.40, 1.40, 3.20, 6.40, 13.20, and 26.40 min, respectively. Each plate was kept covered and exposed to only one fluence of the red light. Survival was determined after the irradiation.

### Effect of MB-aPDT on Bacterial Population

To develop the appropriate MB-aPDT regimen using the minimum effective concentration–fluence dose, we selected two non-lethal MB concentrations, 50 and 150 mg/L to pair with the light fluences of 1, 2, 40, and 80 J/cm ^(2)^.

### Estimation of Post-Treatment Viability and Determination of the Sublethal and Lethal Doses of Treatments

Treated bacterial isolate suspension was transferred from the wells, serially diluted, and streaked on TSA agar plates. The colonies were counted (CFU/ml) after overnight incubation at 37°C. The experiments were performed in triplicates. Sublethal dose was defined as the dose at which the capability of direct damage made by one treatment is not sufficient to destroy most of the bacterial population, whereas the lethal dose was defined as where the extent of direct damage is sufficient to destroy most of the viable bacterial population. For the purpose of the study, sublethal effect was defined as reduction of 0.5–2 log_10_ in CFU/ml and lethal treatment dose defined as reduction of > 2 log_10_ in CFU/ml compared with untreated (Cassidy et al., 2010).

### Statistical Analyses

Comparisons between log_10_ mean changes within the treatment groups and the control were analyzed by using the non-dependent t-test. Data values were expressed as log_10_ means ± standard deviation and mean difference with 95% confidence interval (CI). All statistical analyses were conducted with Stata version 13.1 (StataCorp, College station, Texas, USA). The difference was considered statistically significant when P < 0.05.

## Results

### The Effect of Various MB Concentrations

**Figure 1:**
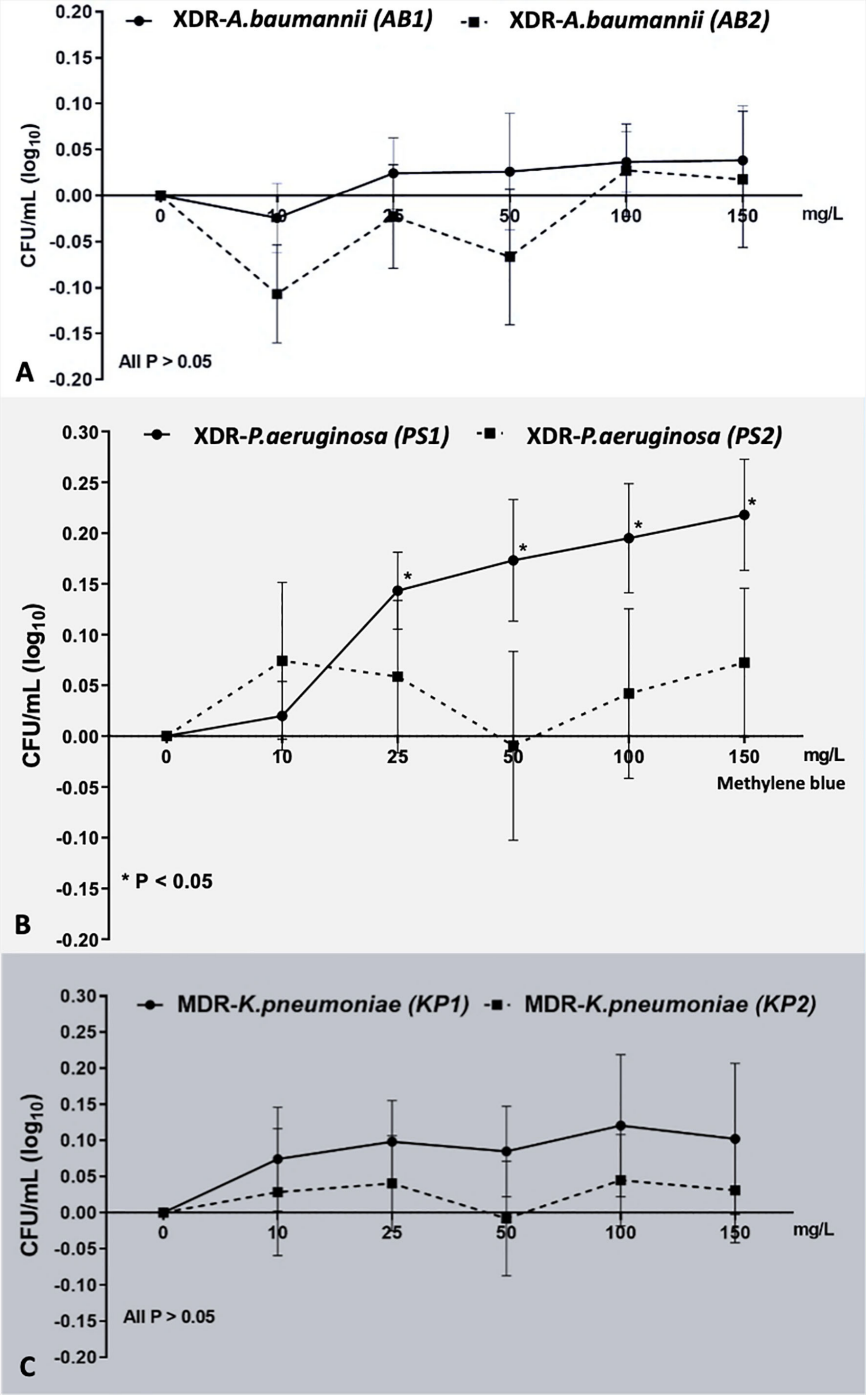
The effect of various MB concentrations on each bacterial strain. MB concentrations could not exhibit the sublethal and lethal effect to all bacterial strains: **(A)** XDR**-**AB1 and AB2, **(B)** XDR**-**PS1 and PS2, and **(C)** MDR-KP1 and KP2. *Statistically significant difference (p-value <0.05).

Apart from the progressively increased survival of PS1 isolates treated with MB of 25, 50, 100, and 150 mg/L (p < 0.05) compared with the control group, there were negligible changes in the viable counts of the bacterial suspensions in the remainder (p > 0.05). None of them demonstrated bactericidal efficacy (**Figure 1**).

### The Effect of Red Light

**Figure 2:**
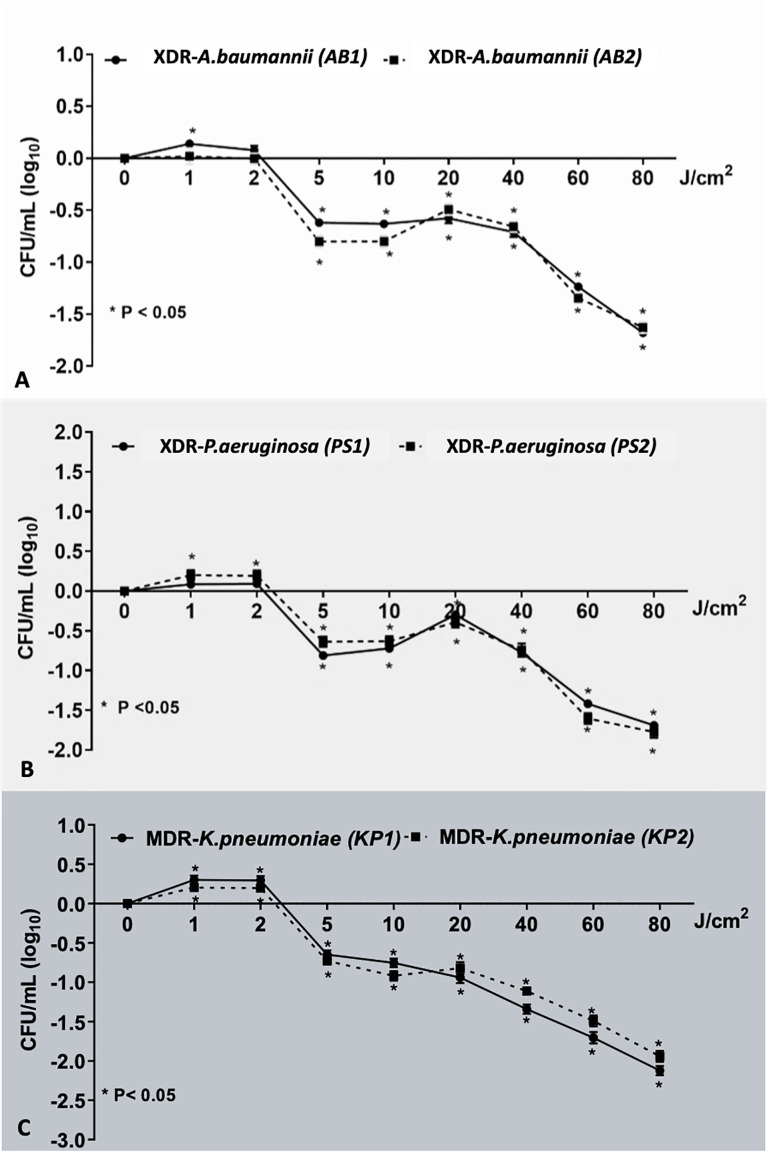
The effect of various red light fluence on each bacterial strain. This demonstrated the effect of red light on bacterial population: **(A)** XDR**-**AB1 and AB2, **(B)** XDR-PS1 and PS2, and **(C)** MDR-KP1 and KP2 in the dose-dependent manner. *Statistically significant difference (p-value <0.05).

There was no reduction of viable bacteria in any isolates at the fluences of 1 and 2 J/cm ^(2)^ of light. At fluences higher than 2 J/cm ^(2)^, reduction trend in viable counts across all the isolates was observed (**Figure 2**). In both strains of AB (AB1 and AB2) and PS (PS1 and PS2) and KP2, sublethal light fluences were demonstrated within the 5 to 80 J/cm ^(2)^ range, which reduced the bacterial viability by up to 1.68, 1.63, 1.69, 1.77, and 1.94 log_10_ units, respectively (p < 0.01). Only for KP1, the lethal dose was achieved with the highest fluence of red light (80 J/cm ^(2)^), which led to viable count reduction by 2.12 (1.96 to 2.28) log_10_ units (p < 0.01). This demonstrated that red light alone had bactericidal effect on certain strains in a fluence-dependent manner.

### MB-aPDT Is an Effective Treatment for Selected XDR and MDR-Gram Negative Bacteria

**Figure 3:**
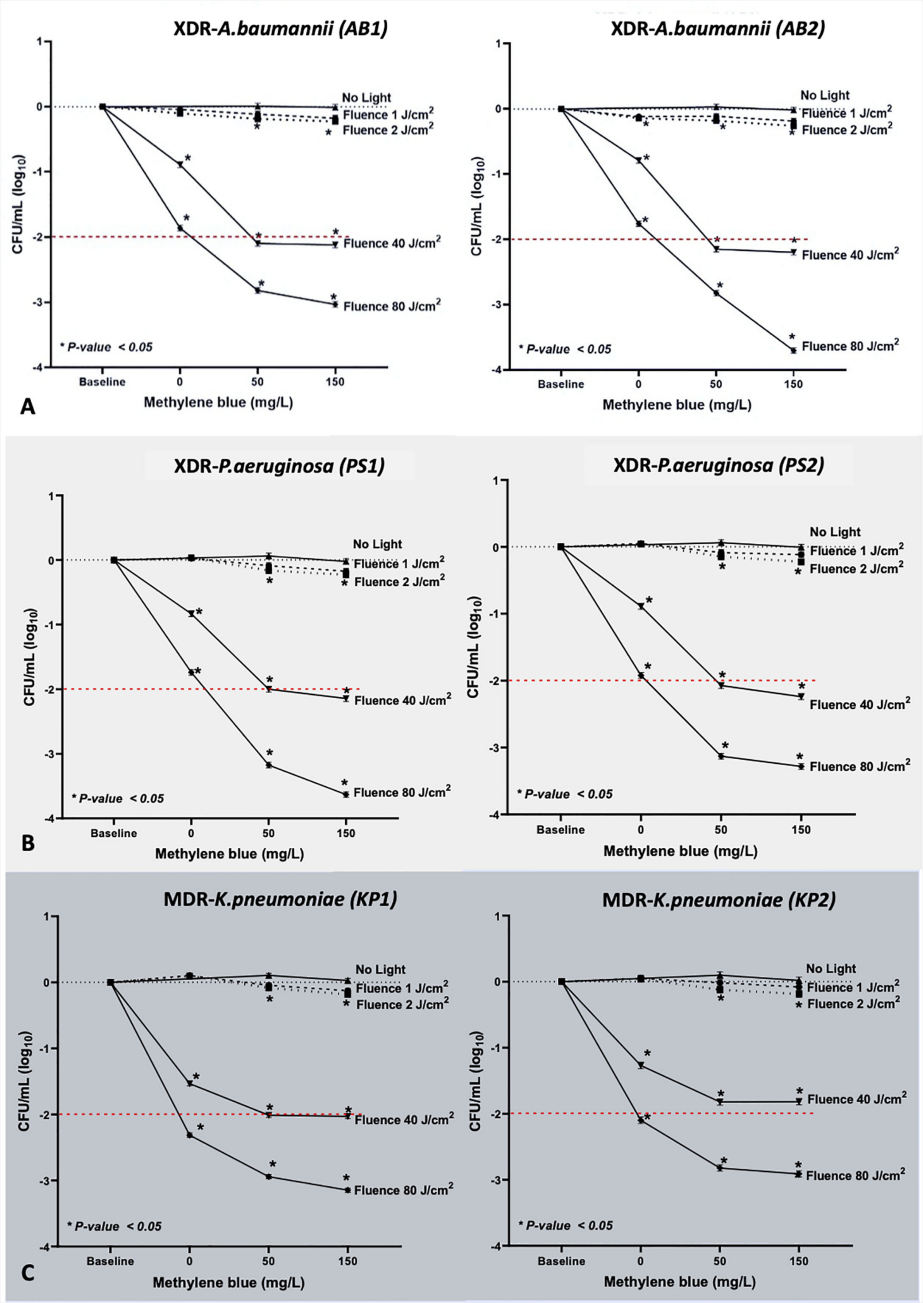
Results of MB-aPDT on **(A) XDR-**AB1 and AB2, **(B) XDR-PS**1 and PS2, and **(C) MDR-**KP1 and KP2, with the light doses of 1, 2, 40, and 80 J/cm^2^ (irradiance of 65 mW/cm^2^, irradiation time from 0.20 to 26.40 min; λ_max_ at 633 nm) and two MB concentrations were tested (50 and 150 mg/L). The values demonstrated were the means of triplicate measurements. *Statistically significant difference (p-value <0.05).

We demonstrated MB-dose/light fluence–dependent antibacterial effects across all the isolates (**Figure 3**). All clinical isolates, for both 50 and 150 mg/L of MB, lethal light fluence was defined because MB of 50 mg/L and 40 J/cm ^(2),^ leading to viable cell reduction over 2 log_10_, except for the PS1 and KP2 as showing only sublethal reduction by 2 (1.91 to 2.09, p < 0.001) and 1.82 (1.73 to 1.91, p < 0.001) log_10_, respectively (**Table 1**). However, when escalating the light fluence to 80 J/cm ^(2)^ with the same concentration, 50 mg/L, the greater bacterial reduction was observed in all clinical isolates, including PS1 and KP2, leading to their lethal viable cell reduction by 3.17 (3.09 to 3.26) and 2.82 (2.73 to 2.91), respectively, and 2.82 (2.72 to 2.91) log_10_ units for AB1 (p < 0.001), 2.83 (2.74 to 2.91) for AB2 (p < 0.001), 3.13 (3.04 to 3.22) for PS2 (p < 0.001), and 2.94 (2.88 to 3.01) log_10_ units for KP1 (p < 0.001). There was statistically significant difference of the viable cell reduction between red light alone and MB-aPDT groups with MB of both 50 and 150 mg/L in all clinical isolates (p < 0.001) (**Table 2**).

**Table 1:** Mean log_10_ difference of MB-aPDT group for XDR-AB, XDR-PS, and MDR-KP compared with baseline.

MB conc.(mg/l)Fluence(J/cm^2^)	0 (control)				50				150			
	XDR-AB1		XDR-AB2		XDR-AB1		XDR-AB2		XDR-AB1		XDR-AB2	
	Mean difference(95% CI)	P-value	Mean difference(95% CI)	P-value	Mean difference(95% CI)	P-value	Mean difference(95% CI)	P-value	Mean difference(95% CI)	P-value	Mean difference(95% CI)	P-value
**0** **(control)**	0.06 (−0.03 to 0.16)	0.20	0.03 (−0.06 to 0.11)	0.54	0.01 (−0.08 to 0.1)	0.85	0.03 (−0.05 to 0.12)	0.47	−0.01 (−0.1 to 0.08)	0.83	−0.02 (−0.1 to 0.07)	0.68
**1**	0.14 (0.05 to 0.23)	0.01	0.02 (−0.20 to −0.24)	0.82	−0.11 (−0.2 to −0.03)	0.01	−0.11 (−0.2 to −0.03)	0.01	−0.18 (−0.26 to −0.09)	<0.001	−0.19 (−0.27 to −0.1)	<0.001
**2**	0.08 (−0.04 to −0.20)	0.14	0 (−0.15 to 0.14)	0.95	−0.19 (−0.28 to −0.09)	<0.001	−0.18 (−0.26 to −0.1)	<0.001	−0.23 (−0.32 to −0.13)	<0.001	−0.26 (−0.34 to −0.18)	<0.001
**40**	−0.71 (−0.84 to −0.58)	<0.01	−0.66 (−0.81 to −0.51)	<0.01	−2.10 (−2.19 to −2)	<0.001	−2.15 (−2.23 to −2.07)	<0.001	−2.12 (−2.21 to −2.02)	<0.001	−2.20 (−2.28 to −2.12)	<0.001
**80**	−1.68 (−1.79 to −1.57)	<0.01	−1.63 (−1.78 to −1.47)	<0.01	−2.82 (−2.91 to −2.72)	<0.001	−2.83 (−2.91 to −2.74)	<0.001	−3.03 (−3.13 to −2.94)	<0.001	−3.70 (−3.79 to −3.62)	<0.001
**MB conc.** **(mg/l)** **Fluence** **(J/cm^2^)**	**0 (control)**				**50**				**150**			
	**XDR-PS1**		**XDR-PS2**		**XDR-PS1**		**XDR-PS2**		**XDR-PS1**		**XDR-PS2**	
	**Mean difference** **(95% CI)**	**P-value**	**Mean difference** **(95% CI)**	**P-value**	**Mean difference** **(95% CI)**	**P-value**	**Mean difference** **(95% CI)**	**P-value**	**Mean difference** **(95% CI)**	**P-value**	**Mean difference** **(95% CI)**	**P-value**
**0** **(control)**	0.05 (−0.04 to 0.14)	0.26	0.08 (−0.01 to 0.17)	0.09	0.06 (−0.03 to 0.15)	0.17	0.06 (−0.03 to 0.15)	0.16	−0.02 (−0.11 to 0.07)	0.59	−0.01 (−0.09 to 0.08)	0.90
**1**	0.09 (−0.01 to 0.19)	0.07	0.20 (0.02 to 0.38)	0.04	−0.09 (−0.18 to 0)	0.05	−0.09 (−0.17 to 0)	0.05	−0.17 (−0.26 to −0.09)	<0.001	−0.12 (−0.2 to −0.03)	0.01
**2**	0.09 (−0.01 to 0.20)	0.07	0.19 (0.01 to 0.38)	0.04	−0.16 (−0.25 to −0.08)	0.001	−0.15 (−0.24 to −0.06)	0.002	−0.23 (−0.32 to −0.14)	<0.001	−0.23 (−0.31 to −0.14)	<0.001
**40**	−0.77 (−0.92 to −0.62)	<0.01	−0.74 (−0.96 to −0.51)	<0.01	−2.00 (−2.09 to −1.91)	<0.001	−2.07 (−2.16 to −1.99)	<0.001	−2.14 (−2.23 to −2.06)	<0.001	−2.24 (−2.33 to −2.15)	<0.001
**80**	−1.69 (−1.79 to −1.59)	<0.01	−1.77 (−1.97 to −1.57)	<0.01	−3.17 (−3.26 to −3.09)	<0.001	−3.13 (−3.22 to −3.04)	<0.001	−3.63 (−3.72 to −3.54)	<0.001	−3.28 (−3.37 to −3.19)	<0.001
**MB conc.** **(mg/l)** **Fluence** **(J/cm^2^)**	**0 (control)**				**50**				**150**			
	**MDR-KP1**		**MDR-KP2**		**MDR-KP1**		**MDR-KP2**		**MDR-KP1**		**MDR-KP2**	
	**Mean difference** **(95% CI)**	**P-value**	**Mean difference** **(95% CI)**	**P-value**	**Mean difference** **(95% CI)**	**P-value**	**Mean difference** **(95% CI)**	**P-value**	**Mean difference** **(95% CI)**	**P-value**	**Mean difference** **(95% CI)**	**P-value**
**0** **(control)**	0.08 (0.01 to 0.15)	0.02	0.04 (−0.05 to 0.13)	0.40	0.11 (0.04 to 0.17)	0.003	0.10 (0 to 0.2)	0.95	0.03 (−0.04 to 0.09)	0.39	0.02 (−0.08 to 0.12)	0.70
**1**	0.30 (0.15 to 0.45)	<0.01	0.20 (0.06 to 0.35)	0.02	−0.04 (−0.11 to 0.02)	0.18	−0.02 (−0.12 to 0.08)	0.72	−0.13 (−0.19 to −0.06)	<0.001	−0.08 (−0.18 to 0.02)	0.10
**2**	0.29 (0.15 to 0.44)	<0.01	0.20 (0.06 to 0.33)	0.02	−0.09 (−0.15 to −0.02)	0.02	−0.12 (−0.21 to −0.03)	0.01	−0.18 (−0.25 to −0.12)	<0.001	−0.19 (−0.28 to −0.1)	<0.001
**40**	−1.34 (−1.50 to −1.18)	<0.01	−1.11 (−1.25 to −0.97)	<0.01	−2.01 (−2.08 to −1.94)	<0.001	−1.82 (−1.91 to −1.73)	<0.001	−2.03 (−2.1 to −1.96)	<0.001	−1.82 (−1.91 to −1.73)	<0.001
**80**	−2.12 (−2.28 to −1.96)	<0.01	−1.94 (−2.14 to −1.75)	<0.01	−2.94 (−3.01 to −2.88)	<0.001	−2.82 (−2.91 to −2.73)	<0.001	−3.15 (−3.21 to −3.08)	<0.001	−2.91 (−3 to −2.82)	<0.001

**Table 2:** Comparison of Mean log_10_ difference between the MB-aPDT and red light alone group for XDR-AB, XDR-PS, and MDR-KP.

Strains Conditions	XDR-AB1		XDR-AB2		XDR-PS1		XDR-PS2		MDR-KP1		MDR-KP2	
	Mean difference (95% CI)	P-value	Mean difference (95% CI)	P-value	Mean difference (95% CI)	P-value	Mean difference (95% CI)	P-value	Mean difference (95% CI)	P-value	Mean difference (95% CI)	P-value
F40	Ref	Ref	Ref	Ref	Ref	Ref	Ref	Ref	Ref	Ref	Ref	Ref
F40/MB50	−1.21 (−1.35 to −1.07)	<0.001	−1.36 (−1.49 to −1.23)	<0.001	−1.17 (−1.28 to −1.05)	<0.001	−1.18 (−1.30 to −1.06)	<0.001	−0.48 (−0.56 to −0.40)	<0.001	−0.55 (−0.65 to −0.46)	<0.001
F40/MB150	−1.23 (−1.37 to −1.09)	<0.001	−1.41 (−1.53 to −1.28)	<0.001	−1.31 (−1.43 to −1.19)	<0.001	−1.35 (−1.47 to −1.23)	<0.001	−0.5 (−0.58 to −0.42)	<0.001	−0.55 (−0.65 to −0.45)	<0.001
F80	Ref	Ref	Ref	Ref	Ref	Ref	Ref	Ref	Ref	Ref	Ref	Ref
F80/MB50	−0.96 (−1.08 to −0.84)	<0.001	−1.06 (−1.14 to −0.99)	<0.001	−1.43 (−1.56 to −1.31)	<0.001	−1.21 (−1.37 to −1.04)	<0.001	−0.63 (−0.70 to −0.56)	<0.001	−0.72 (−0.86 to −0.59)	<0.001
F80/MB150	−1.17 (−1.29 to −1.05)	<0.001	−1.94 (−2.02 to −1.87)	<0.001	−1.89 (−2.02 to −1.76)	<0.001	−1.36 (−1.53 to −1.19)	<0.001	−0.83 (−0.91 to −0.76)	<0.001	−0.81 (−0.95 to −0.68)	<0.001

Ref, reference/baseline; F, red light fluence (J/cm^2^); F40, red light fluence at 40 J/cm^2^; F80, red light fluence at 80 J/cm^2^; MB, methylene blue; MB50, methylene blue of 50 mg/L; MB150, methylene blue of 150 mg/L.

## Discussion

Infections in this drug resistance era further complicate wounds causing an enormous hurdle to overcome (Mulani et al., 2019). We demonstrated the promising outcomes of MB-aPDT against clinically important isolates of XDR-AB, XDR-PS, and MDR-KP from Thailand of which pathogens WHO prioritized research and development of new treatments (Medina and Pieper, 2016; Mulani et al., 2019). Looking at the reduction pattern of the bacterial viability after MB-aPDT (**Figure 3**), although MDR-KP appeared to be less resistant regarding the antimicrobial susceptibility pattern profiles, XDR-AB and XDR-PS generally seemed more susceptible to MB-aPDT than MDR-KP. This differential sensitivity is not, by any means, unexpected. Importantly, there were independent patterns of the aPDT susceptibility of the clinical isolates regardless of the patterns of antimicrobial resistance (**Supplementary Table 2**), emphasizing its known exclusive non-selective mechanism of action (Liu et al., 2015). The major advantage of PDT lies in the fact that, with appropriate fluences of light and oxygen, PSs undergo an intersystem crossing to generate triplet state oxygen molecules ( ^(3)^O_2_). Reactive oxygen species (ROS), such as singlet oxygen, superoxides, and hydroxyl radicals, have a broad-spectrum effect on numerous microbial targets (Vatansever et al., 2013). This unique mechanism of action constitutes by default bactericidal effects independent of antimicrobial resistance patterns (Tegos and Hamblin, 2006; Tegos et al., 2008; Huang et al., 2012; Huang et al., 2012; Liu et al., 2015).

One beauty of PDT is one can vary PSs’ concentrations as well as light fluences, and even frequency of the treatment to tackle different pathogens. Many PSs possess unique properties. With different PSs, the effective antimicrobial properties were reported to be considerably variable in the same settings of irradiation against the same bacterial strains (Merigo et al., 2019). We particularly chose MB due to its several advantages over other PSs. The cationic charge of non-toxic MB, a derivative of phenothiazinium dyes, provides high binding affinity to multiple microbial targets, e.g., cell membrane, mitochondria, and nucleic acids (Zeina et al., 2001; Kashef et al., 2011; Vatansever et al., 2013; Liu et al., 2015). Moreover, MB is an “instant” PS, requiring no incubation period, allowing significantly shorter treatment time along with its hydrophilic polarity property rendering it a painless treatment (Castano et al., 2004; Bowornsathitchai et al., 2020). MB is also a pragmatic choice for use in most healthcare settings in which neither commercial PSs nor in-house preparation are available. In brief, MB solution for intravenous administration, which is widely available across the world, can be a viable and economical option (Kashef et al., 2011; Kawczyk-Krupka et al., 2018).

The selection of light fluences can be rather challenging as different effects have been observed, also in our study. This can be explained by biphasic biological response curve of visible light–based treatments, particularly in the red to near-infrared region whereby too low or too high fluences can lead to unwanted effects (Huang et al., 2009; Huang et al., 2011). When appropriate fluences are used, photobiomodulation (PBM) leading to photoactivation of endogenous molecules, particularly cytochrome C oxidase, subsequently activates the intracellular ROS generation, of which certain high levels may potentially cause cell damage (Huang et al., 2011; Kashef et al., 2011; Wozniak et al., 2019). This might explain why higher fluences of red light alone, 5 to 80 J/cm ^(2)^ in this study, generally reduced the bacterial population to the sublethal and lethal point. Although the lethal light dose, 80J/cm^2^ was achieved for KP1 without MB combined, to find the minimum effective concentration–fluence dose of MB-aPDT, we then performed the similar combinations of concentration–fluence doses to what were used for the other selected pathogens resulting in the minimum effective dose of MB-aPDT for KP1 of MB of 50 mg/L and light of 40 J/cm^2^.

Alongside with partial antibacterial effect, PBM can also activate multifaceted growth factors, especially extracellular transforming growth factor β, which has the crucial role for assisting wound repair. Moreover, PBM can also promote regeneration of skin appendages, epithelial migration and proliferation, endothelial migration for angiogenesis, fibroblast matrix synthesis, and wound contraction.Mosca et al., 2019 Taken together, PDT seems very promising both as monothrapy (Huang et al., 2012; Huang et al., 2012; Sperandio et al., 2013; Longo et al., 2014; Liu et al., 2015) and as an adjuvant treatment in chronic wound condition, with wide range of anti-microbial activities, especially upon otherwise drug-resistant lethal strains, and as wound healing enhancer (Gollnick et al., 2003; Weber et al., 2012; Reginato et al., 2014; Anzengruber et al., 2015; Corsi et al., 2016; Grandi et al., 2018; Oyama et al., 2020; Sun et al., 2020).

The optimum effect of MB-aPDT was also related to other factors, for example, the internalization of PS into the bacterial cell and the bacterial protective mechanism (Huang et al., 2012; Longo et al., 2014). MDR and XDR gram-negative bacteria have a unique mechanism to encounter the harmful substances by using the transmembrane proteins called multidrug resistance pumps such as AcrAB-TolC, one of the tripartite pumps expressed in *Escherichia coli*, MexAB-OprM in *P. aeruginosa (* Lewis, 1999 *)*, and *kpnGH* in *K. pneumoniae (* Srinivasan et al., 2014 *)*, that manage the efflux of amphipathic cations outside the cells, including MB. Efflux pump inhibitors (EPIs) (Tegos et al., 2008) as seemingly promising strategies have been intensely researched because the first multidrug efflux pump was identified in 1996 (Paulsen et al., 1996) yet approved for clinical use (Ughachukwu and Unekwe, 2012). Some studies suggested using electron acceptor, such as sodium azide and potassium iodide (Vecchio et al., 2015), to potentiate the aPDT outcome when MB-aPDT yields suboptimal results (Huang et al., 2012). Furthermore, certain organisms have distinctive mechanisms to protect them from deleterious exogenous causes. For example, AB, PS, and KP have an adroit capacity to rapidly adapt themselves from planktonic to biofilm phase and quorum sensing against the injury (Longo et al., 2014). These might somehow explain the different number of reductions in our study when using the same MB-aPDT setting on each bacterial strain.

In addition, sublethal and lethal parameters of aPDT also affect the frequency and duration of aPDT protocol. Recent studies showed that repetitive uses of sublethal doses of aPDT potentially lead to tolerance adaptation (Leanse et al., 2018; Pieranski et al., 2020) of bacteria through multiple metabolic pathways, e.g., cell wall biogenesis, DNA recombination/repair (Kashef and Hamblin, 2017), and a stimulation of biofilm formation (Schembri et al., 2003; Murphy et al., 2005; Wen et al., 2005; Villa et al., 2012; Hendiani et al., 2019). This situation is dissimilar to the mechanism of bacterial resistance in which the bacteria are still eliminated but require more frequency and higher light fluences. Thus, antimicrobial photoinactivation essentially needs precise application of the lethal doses of MB and red light combined to minimize this phenomenon. Our suggested lethal concentration–fluence dose of 50 mg/L with 80 J/cm ^(2)^ from this study might be useful as a starting reference for clinical application and further development of an optimal aPDT protocol.

Other aPDT protocols against ESKAPE pathogens employing different PSs and lights have been published both as *in vitro* and *in vivo* studies (Dai et al., 2009; Huang et al., 2014; Maisch et al., 2014; Zhang et al., 2014; Boluki et al., 2017; Khan et al., 2017; Yuan et al., 2017; Pereira et al., 2018; Yang et al., 2018; Hendiani et al., 2019; Marcolan De Mello et al., 2019a; Wozniak et al., 2019). Most PSs used were of phenothiazinium-based dyes, including MB, Rose Bengal (RB), and toluene blue O (TBO). Similar to our study yet using one concentration of MB coupled with variable fluences and step ranges of red light against a wide range of global priority drug-resistant bacteria including ESKAPE pathogens and two yeast species, *Candida albicans* and *Cryptococcus neoformans*, Sabino CP et al. (Huang et al., 2012; St Denis et al., 2013; Sarker et al., 2021) successfully demonstrated the efficacy of MB-aPDT against across all the selected strains in species-specific dose–response kinetics regardless of drug-resistance profiles. The improvement of the efficacy of MB-aPDT was observed with the addition of some chemical compounds (Vecchio et al., 2015) including an inert inorganic salt potassium iodide (KI) (Vecchio et al., 2015), which seems to be safe for future clinical application. With different combined additives, Sarker et al. (Ishiwata et al., 2021) have recently reported the efficacy of MB-aPDT with three once-daily treatment sessions in controlling burn wounds infected with PS from the wound surface to the region deeper than 1,200 μm in the hypodermis in rats. Ishiwata et al. (2021) have demonstrated the importance of repetitive MB-aPDT application, which was given every 24 h for 7 consecutive days for controlling bacterial migration and extending the survival of rats with an extensive deep burn wound infected with PS. Wozniak et al. (2019) reported aPDT using RB, coupled with green light (λ_max_, 515 nm) on XDR-AB. In another study De Mello et al. performed MB-aPDT against MDR-AB (Marcolan De Mello et al., 2019b). A study by Boluki et al. (2017) demonstrated successful bactericidal activity of sublethal aPDT using TBO, with red light–emitting diode (λ_max_, 630 nm) in a combination with colistin against PDR-AB *(* Park et al., 2011 *).* Moreover, in addition to the efficacy of aPDT against bacteria, PDT is also a powerful weapon against superficial mycoses, either dermatophytes or non-dermatophytes. Recent systematic review showed that MB-PDT is as efficacious if not superior to ALA and MAL when treating onychomycosis (Shen et al., 2020).

The strengths of our study are that, first, we demonstrated the effects of MB in various concentrations, red light, and the combination of the two, and MB-aPDT against locally isolated XDR-AB, XDR-PS, and MDR-KP. All the strains used were all clinical isolates that should well represent pathogens of clinical concerns particularly in our local region. However, there were some limitations worth mentioning here. The bacterial strains utilized in the study were isolated from various settings rather than entirely from chronic wounds. Second, the process of PS incorporation into the bacterial cells in the laboratory experiments might be different from actual clinical scenarios. Third, we used only MB and red light in our study. Other PSs and light sources should be investigated in the future. Last, we investigated “single” PDT regimen, while in fact, the painless nature and short-irradiation protocol may allow for repeat treatments, adding even further benefits in clinical settings.

## Conclusion

MB-aPDT evidently killed the selected XDR and MDR-gram negative bacteria. In a highly drug-resistant crisis era, MB-aPDT could be one of the promising options, particularly for local infections and infection complicating chronic wounds with one suggested combination of MB of 50 mg/L and red light of 80 J/cm ^(2)^ of red light as a guide to begin with. Both *in vitro* study for other MDR-bacteria and *in vivo* study to establish precise PDT protocol for effective antimicrobial and wound healing purposes are to be investigated.

## Data Availability Statement

The original contributions presented in the study are included in the article/**Supplementary Material**. Further inquiries can be directed to the corresponding author.

## Ethics Statement

No human studies are presented in the manuscript. However, the study protocol was approved by the Institutional Review Board of Faculty of Medicine, Chulalongkorn University.

## Author Contributions

EB contributed to conception, design of the study, resources, manuscript editing and revision, supervision, and submission. CS organized the database, performed the statistical analysis, and wrote the first draft of the manuscript. PA supervised and edited the manuscript. JV shared resources. TC supervised laboratory tasks. All authors contributed to the article and approved the submitted version.

## Funding

The study was supported by Ratchadapisek Sompoch Endowment Fund, RA 63/086, Faculty of Medicine, Chulalongkorn University.

## Conflict of Interest

The authors declare that the research was conducted in the absence of any commercial or financial relationships that could be construed as a potential conflict of interest.

## Publisher’s Note

All claims expressed in this article are solely those of the authors and do not necessarily represent those of their affiliated organizations, or those of the publisher, the editors and the reviewers. Any product that may be evaluated in this article, or claim that may be made by its manufacturer, is not guaranteed or endorsed by the publisher.
