# Supplementary material for: Methylene Blue–Mediated Antimicrobial ​Photodynamic Therapy Against Clinical Isolates of Extensively Drug Resistant ​Gram-Negative Bacteria Causing Nosocomial Infections in Thailand, An In Vitro Study
Source: Front Cell Infect Microbiol. 2022 Jul 1;12:929242. doi: 10.3389/fcimb.2022.929242 (PMC9283779; doi:10.3389/fcimb.2022.929242)

Supplementary Table 1 Detailed patients’ profiles of each isolate

| Isolates No. | Specimens | Location/purpose | Collection  date | Patient profile | | Patient status/ Medical background |
| --- | --- | --- | --- | --- | --- | --- |
|  |  |  |  | Sex | Age (year) |  |
| XDR-AB 01 | Sputum | Surveillance sputum culture | 9.6.2020 | Male | 57 | Liver cirrhosis classified as Child A due to chronic hepaititis C infection 2. Hepatocellular carcinoma classified as BCLC stage B with minimally invasive intraductal paillary neoplasm of the bile duct S/P Rt. trisectionectomy |
| XDR-AB 02 | Pus, Wound | Chronic erosive wound, Rt leg | 9.6.2020 | Female | 1 | Epidermolysis bullosa (KRT5 mutation) |
| XDR-PS 01 | Bile | Percutaneous transhepatic biliary drainage malfunction with acute cholangitis | 8.6.2020 | Male | 80 | 1. Unresectable hilar cholangiocarcinoma S/P Percutaneous transhepatic biliary drainage (PTBD) with previous multiple episodes of PTBD malfunction 2. Lower urinary tract symptoms with recurrent urinary tract infections 3. Hashimoto thyroiditis |
| XDR-PS 02 | Urine | Urinary tract infection  with septic shock | 6.5.2020 | Male | 74 | 1. The bed-ridden with Parkinson's disease 2. Previous multiple episodes of recurrent pneumonia S/P tracheostomy |
| MDR-KP 01 | Sputum | Pneumonia | 8.6.2020 | Male | 92 | 1. Chronic kidney disease on regular hemodialysis 2.Rt foot osteomyelitis with septic shock 3. Tracheobronchomalacia with A.baumannii colonization |
| MDR-KP 02 | Sputum | Aspiration pneumonia | 16.5.2019 | Male | 94 | The bed-ridden with aspiration pneumonia  with sepsis S/P tracheostomy |

Supplementary Table 2 Detailed antimicrobial susceptibility pattern of each isolate according to CLSI guideline^25^


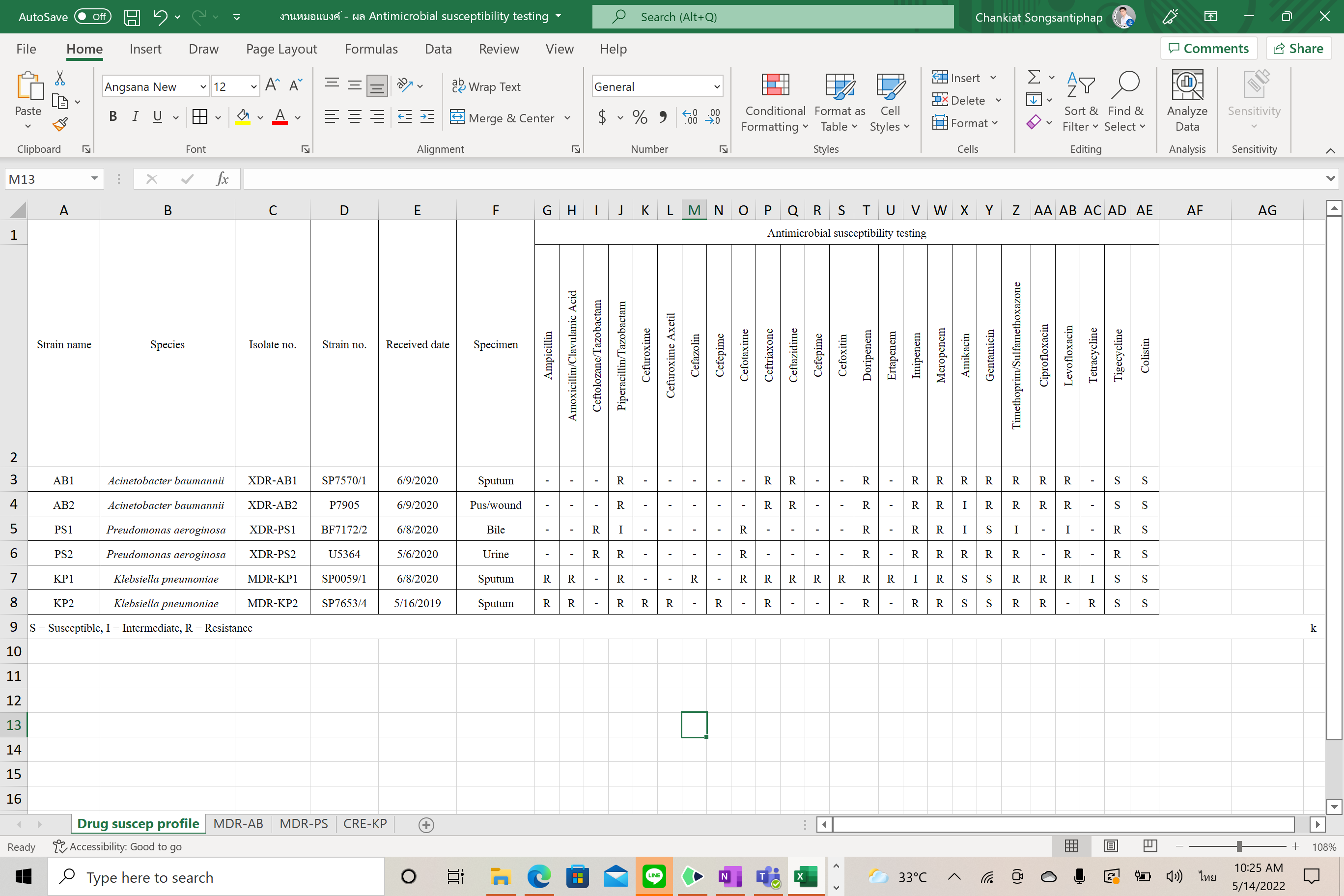

Supplement: Supplementary file 1 [file Table_1.docx]
